# Air Pollution and Heat Impacts on Respiratory Morbidity and Mortality Outcomes in Africa: A Systematic Review Towards a Meta-analysis

**DOI:** 10.1007/s40572-026-00561-7

**Published:** 2026-09-03

**Authors:** Caradee Y Wright, Thandi Kapwata, Viwe Dikoko, Chantelle Howlett-Downing, Natasha Naidoo

**Affiliations:** 1 Environment and Health Research Unit, South African Medical Research Council, SAMRC, 1 Soutpansberg Road, Pretoria, South Africa; 2 Department of Geography, Geoinformatics and Meteorology, University of Pretoria, Pretoria, South Africa; 3 Environment and Health Research Unit, South African Medical Research Council, Johannesburg, South Africa; 4 African Climate and Development Initiative, University of Cape Town, Cape Town, South Africa; 5 School of Health Systems and Public Health, Faculty of Health Sciences, University of Pretoria, Pretoria, South Africa; 6 Environment and Health Research Unit, South African Medical Research Council, Durban, South Africa

**Keywords:** Climate change, Environmental health, Policy, Preparedness, Public health (*n* = 6 max)

## Abstract

**Purpose of the review:**

This review synthesised evidence on associations between air pollution and respiratory morbidity in Africa. Following PRISMA guidelines, we systematically searched PubMed, ScienceDirect and Elicit for case-control studies published between 2015 and 2025.

**Recent findings:**

Thirteen studies from ten African countries reported pollutant levels far exceeding WHO guidelines. Indoor PM₂.₅ in biomass-using homes ranged from 96 to 177 µg/m³, and ambient PM₂.₅ reached 259 µg/m³. Nitrogen oxides were consistently associated with reduced lung function in children, while household air pollution increased risks of under-five mortality and low birthweight. Associations with acute respiratory infections varied across settings. Vulnerability was greatest among young children, those with airway hyperresponsiveness, and households with poor ventilation. Only three studies included temperature, and none examined heat–respiratory interactions.

**Summary:**

Across African case-control studies, particulate matter and household air pollution remain consistently linked to adverse respiratory outcomes, highlighting urgent needs for cleaner fuels, improved ventilation, and stronger evidence on combined pollution and heat exposures.

**Supplementary Information:**

The online version contains supplementary material available at https://doi.org/10.1007/s40572-026-00561-7.

## Introduction

Air pollution is a leading environmental risk factor for global morbidity and mortality, with low- and middle-income countries bearing a disproportionate share of the health burden [1]. In Africa, rapid urbanisation, reliance on biomass fuels, expanding transport emissions, and industrial activity contribute to some of the highest ambient and household pollution levels worldwide [2]. At the same time, respiratory diseases remain major causes of illness and death across the continent, particularly among children, older adults, and individuals with underlying conditions [3]. Despite these intersecting vulnerabilities, evidence describing pollution–respiratory health associations in African populations remains limited and fragmented, with few studies employing robust epidemiological designs [4].

Understanding these associations is essential for informing policies that address energy transitions, air-quality management, and public health planning. While global literature [5] has established clear links between particulate matter, nitrogen oxides, and a range of respiratory outcomes, it is unclear whether these patterns hold consistently in African settings, where exposure profiles, household behaviours, climate conditions, and health system contexts differ substantially. Recent studies have begun to explore these relationships, yet findings remain heterogeneous and often constrained by small sample sizes, limited exposure assessment, and variable study quality [6]. This systematic review synthesises current case-control evidence to clarify respiratory risks associated with air pollution in Africa and identify priorities for future research and policy action.

## Methods

### Study Design and Objective

This systematic review was designed to synthesize evidence from case–control studies conducted in sub-Saharan Africa between 2015 and 2025 that examined the relationship between air pollution and respiratory morbidity outcomes. The review aimed to (1) quantify the magnitude and direction of associations between these exposures and respiratory outcomes, (2) identify population groups most vulnerable to combined environmental stressors and (3) conduct a meta-analysis from the included studies.

### Literature Search Strategy

A comprehensive literature search was conducted on the PubMed and ScienceDirect databases and supplemented using the Elicit semantic search engine.

### Screening and Eligibility Criteria Using

Studies were screened using Population, Exposure, Comparison, Outcome, Study Design (PECOS) criteria with strict inclusion criteria to ensure methodological rigor and relevance (Table 1). Geographic scope was limited to African countries or multi-country analyses that included at least one African setting. Exposure assessments focused on air pollutants (e.g., PM₂.₅, NO₂, CO, black carbon). Studies reporting respiratory outcomes such as acute respiratory infections, pneumonia, chronic obstructive pulmonary disease, and asthma, birth weight and mortality, were considered. Quantitative analyses had to include effect estimates linking exposure to health outcomes, and only peer-reviewed articles published between 2015 and 2025 were included. Full-text availability was required for quality appraisal. Studies failing to meet these criteria, including reviews or those conducted outside Africa, were excluded (Table 1).

**Table 1:** The inclusion and exclusion criteria for screening according to the PECOS framework

PECOS Framework	Inclusion	Exclusion
Population	Human populations of any age group in **African countries**	Studies not conducted in Africa; animal or in-vitro studies
Exposure and comparator	• Ambient (outdoor) or household air pollution: PM_2.5_, PM_10_, NO₂, SO₂, O₃, CO, black carbon, VOCs, AQI, etc. • Short-term or long-term exposure acceptable Comparators: Lower exposure level, different temperature/climate strata, or statistical reference value as reported by study	• Pollutant exposure without a respiratory outcome • Indoor pollution unrelated to household environment only (e.g., specific workplace chemical exposures) • Modelling/projection studies without observed epidemiological data
Outcomes	• Respiratory morbidity (e.g., asthma exacerbations, COPD, pneumonia, low-birth weight, hospital admissions, emergency visits) • Respiratory mortality (all cause, cardiovascular and respiratory, ICD-based or author-defined) • Quantitative effect estimates (OR, HR, % change, regression coefficients) with CI or sufficient data for calculation	• Non-respiratory outcomes only • Studies reporting only air quality or exposure without health outcomes • Birth-only outcomes (e.g., low birth weight, pre-term birth, stillbirth) unless respiratory-related
Setting	Conducted in **any African country** (urban or rural)	Conducted outside Africa
Type of study design	Case-control studies	Quantitative study designs other than case-control studies; reviews; protocols; purely laboratory or toxicological studies
Type of paper	Peer-reviewed original research manuscripts	Grey literature; conference abstracts; case reports; editorials; commentaries
Language	English (or other languages if translation available)	Unavailable language translations
Time period	2015–2025	Manuscripts more than 10 years old

### Data Extraction

Data were extracted using structured templates applied by the Elicit large language model trained for systematic review tasks. Variables extracted included: Study characteristics: Country, setting (urban/rural), and participant demographics (age, sex, sample size); Exposure assessment: Type of pollutant(s), measurement method (monitoring, modelling, or survey), temporal averaging period, and indoor/outdoor classification; Outcome definition: Disease category, diagnostic criteria, and data source (hospital, survey, or registry); Effect estimates: Odds ratios and confidence intervals; and Confounder control: Adjustment for age, sex, socioeconomic status, smoking, weather, and other relevant variables.

### Data Synthesis and Analysis

Extracted data were narratively synthesized because of heterogeneity in exposure metrics, outcome definitions, and statistical modelling approaches. Studies were grouped by pollutant type (particulate, gaseous, household), exposure setting (indoor vs. ambient), and population subgroup (children, adults, women). Quantitative summaries were used where possible to highlight ranges of effect estimates and confidence intervals. Emphasis was placed on identifying patterns of risk, and geographic or socioeconomic differentials across studies.

### Quality Appraisal

Methodological quality and risk of bias were appraised holistically, considering study design rigor, exposure measurement accuracy, control selection, confounder adjustment, and completeness of statistical reporting. Studies with unclear methods, missing data, or lacking control for key confounders were noted as limitations in the synthesis.

## Results

### Descriptive Findings

**Fig. 1:**
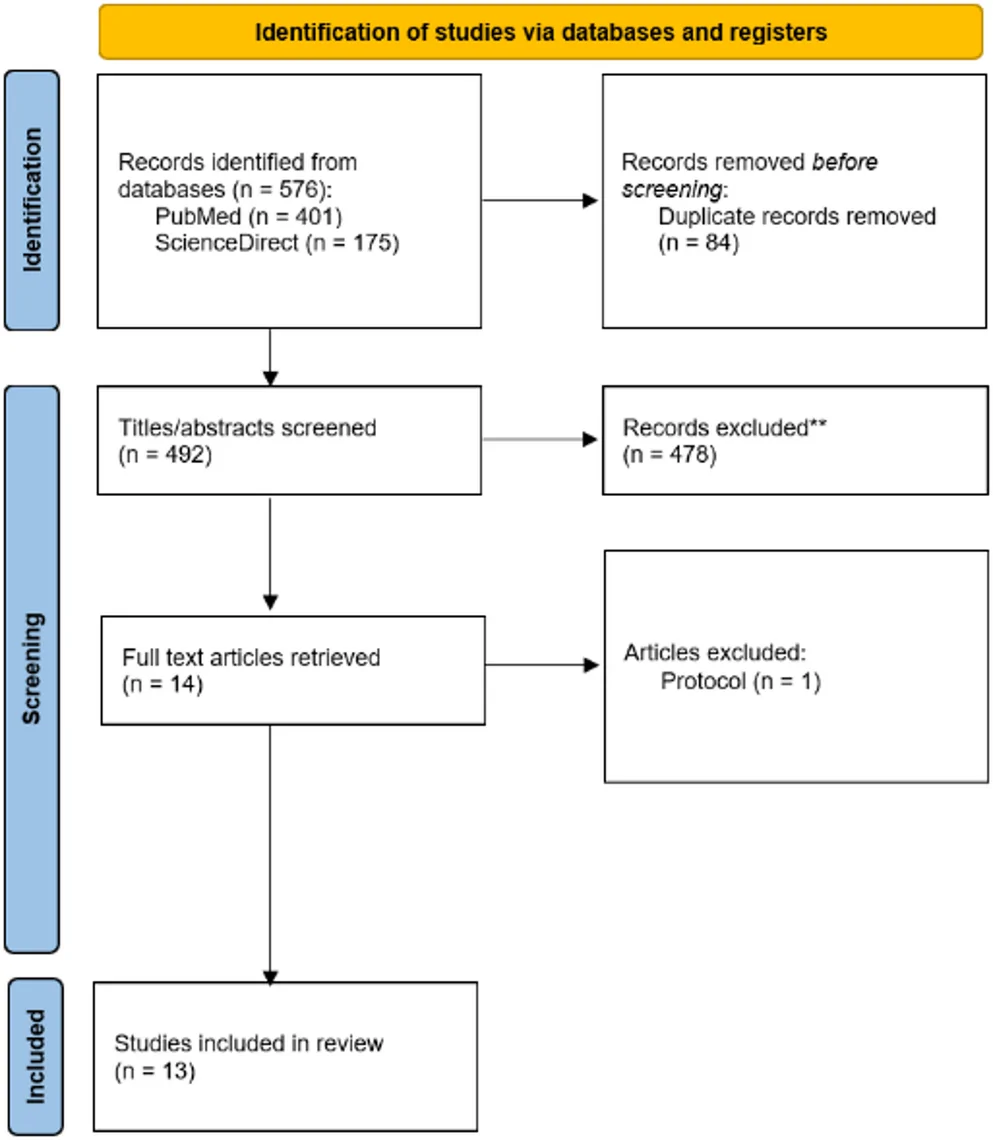
PRIMSA flow diagram showing the study selection process

The review identified 13 studies (Fig. 1; Table 2) examining associations between air pollution and respiratory outcomes in Africa, conducted across 10 countries in sub-Saharan Africa. Studies varied substantially in design, population, exposure assessment, and outcomes measured. Studies employed diverse exposure assessment methods. Ambient air pollution was measured using monitors for PM_2.5_, PM_10_, NO_2_, NO, and SO_2_. Household air pollution (HAP) exposure was primarily assessed through cooking fuel type (biomass vs. clean fuels) or direct measurement of indoor pollutants. One study uniquely examined airborne bacterial and fungal microbiota [7]. Temperature was measured in several studies, with ranges from 17℃ to 33℃ in Burundi [8] and 25–30℃ in Kenya [9], though most studies did not report temperature as a primary exposure.

**Table 2:** Description of each study included in the systematic review

First author, year of publication	Country	Setting	Study Design	Population; sample size	Study period	Pollutant	Outcome	Effect estimate; statistical significance	Key findings
Abereton, 2025	Nigeria, Niger Delta	Rural / industrial (crude-oil impacted)	Cross-sectional	Children < 5 yrs; *N* = 450	Jul-Oct 2022	PM_2.5_, PM_10_, TVOCs, and HCHO	Respiratory health symptoms (e.g., cough, difficulty breathing, persistent nasal congestion)	ORs ranging from 2.53 to 14.18 for various respiratory symptoms (*p* < 0.001)	Mean concentrations of pollutants were consistently higher and frequently exceeded WHO national standards in crude oil-impacted communities. Children in these communities had a significantly higher prevalence and likelihood ORs of experiencing respiratory symptoms compared to those in a less-impacted community.
Adaji, 2020	Nigeria, Abuja	Urban, rural	Case-control	Children < 5 yrs; *N* = 290 (150 cases, 140 controls)	Jan 2018-Jan 2019	Indoor Air Pollutants: Black Carbon PM_2.5_, PM_1_, Carbon Monoxide (CO)	Pneumonia episodes	BC = 0.0260 (*p* < 0.05) BC was higher in cases − 4350 µg/m^3^ than controls − 4126 µg/m^3^ PM_2.5_: Significant differences at specific exposure durations − 10, 15, 20–24 h (*p* < 0.05).	BC exposure was positively and significantly associated with a pneumonia episode in children under five. PM_2.5_ also showed significant differences in exposure duration between cases and controls. Other factors, including children being present during cooking, number of available windows, and MUA (Mid-Upper Arm Circumference), were also found to increase the likelihood of a pneumonia episode.
Demelash, 2015	Ethiopia, Bale zone	Urban, rural	Case-control	Mothers with singleton, full-term births; *N* = 387 (136 cases, 272 controls)	April-Aug 2013	Intimate Partner Violence during pregnancy (Physical, Sexual, or Emotional)	Low Birth Weight	aOR = 3.00 (95% CI 1.57 to 7.18). (*p* < 0.05).	Mothers who suffered any type of Intimate Partner Violence during pregnancy were three times more likely to have a newborn with Low Birth Weight compared to mothers who were not abused. Intimate Partner Violence was reported by 25.8% of all mothers interviewed, but 48% of mothers with low birth weight infants reported abuse.
Fakunle, 2023	Nigeria, Ibadan	Urban	Case-control	Children < 5 yrs; *N* = 197 (98 cases, 99 controls)	Mar 2019-Feb 2020	Indoor Airborne Microbial Communities: Bacterial (16 S rRNA gene) and Fungal (ITS region-1)	Lower Respiratory Tract Infections (LRTI)	Bacterial richness (OR 1.06/100 units) and Shannon diversity (OR 1.92/unit) increased LRTI odds. Deinococcota and Bacteroidota were negatively, and **Ascomycota** positively, associated with LRTI (*p* < 0.005–0.001).	Increased bacterial richness and diversity in house dust were independently associated with a higher likelihood of childhood LRTI. Bacterial and fungal community composition differed significantly between cases and controls. The findings suggest that early-life exposure to specific airborne bacterial and fungal communities is associated with LRTI development.
Hashazimari, 2022	Burundi, Bujumbura	Urban, industrial	Case-control	All ages; *N* = 344 935 (117 812 exposed, 227 123 unexposed)	2018–2020	Contaminants spread from an unprotected landfill (including viruses and bacteria via air from polluted soil)	Acute Respiratory Infections (ARI)	OR 3.19 and RD 19.6%, indicating a statistically significant increased risk (per MH test).	There was a significant excess risk of contracting ARI in the area exposed to the unprotected landfill. The risk decreased the further away the population was from the landfill. Children under 5 years of age were more affected compared to those over 5 years of age.
Jary, 2017	Malawi, Blantyre	Urban	Case-control	Adults (145 cases, 253 controls); *N* = 398	Jul 2014-Feb 2016	Household air pollution (PM, CO)	Radiologically confirmed pneumonia in adults (by HIV status)	PM aOR (HIV-positive): 1.00 (95% CI 1.00–1.01, *p* = 0.141) • PM aOR (HIV-negative): 1.00 (95% CI 0.99–1.01, *p* = 0.872)	HAP exposure (PM and CO) was not associated with pneumonia in Malawian adults, regardless of HIV status. The child–HAP association did not appear in adults. Chronic respiratory disease was a strong independent predictor (aOR 28.07–104.27).
Benoit, 2019	Burkina Faso, Ouagadougou	Urban	Case-control	Children < 5 yrs; *N* = 371 (125 cases, 250 controls)	Sep-Oct 2018	PM₂._5_ from biomass cooking	Acute respiratory infections (ARI)	ORs are not reported, but association described as statistically significant	Indoor biomass use was significantly associated with ARI in young children. PM₂.₅ levels were very high (above WHO guidelines). Having more bedrooms was protective, suggesting improved ventilation reduces ARI risk.
Mbeje, 2022	South Africa, KwaZulu-Natal	Urban	Case-control	Adults; *N* = 234 (75 cases, 159 controls)	Jan 2018-Mar 2019	Tobacco smoking, passive smoke, alcohol use, and history of lung disease	Lung cancer	Smoking: OR 2.86 (95% CI 1.21–6.77) • Passive smoke: OR 3.28 (95% CI 1.48–7.30) • Alcohol: aOR 2.79–3.35 • Prior lung disease: aOR 9.91–12.1 (All significant)	Active and passive tobacco smoke were strong risk factors for lung cancer. Environmental and occupational carcinogens, alcohol use, and pre-existing lung disease also markedly increased lung cancer risk.
Bickton, 2020	14 sub-Saharan African countries	Predominantly rural	Secondary analysis of District Health data	Children < 5 yrs (*N* = 164 376)	2015–2018	Household air pollution (biomass fuel for cooking)	Under-five mortality	aOR 1.33 (95% CI 1.03–1.71), significant; Cooking location ORs: Separate building 0.85, Outside 0.75	HAP from biomass cooking increased under-five mortality risk. Cooking inside the home posed higher risk than cooking in a separate building or outdoors. Breastfeeding was strongly protective.
Mentz, 2019	South Africa, Durban	Urban, industrialized	Panel study	Schoolchildren (*N* = 423)	Jul 2004-Feb 2005	NO, NO₂, SO₂, PM₁₀	Daily lung function changes (FEV₁) in school children	NO: 13–18 mL decline per IQR; NO₂: 14–23 mL per IQR; 5-day NO₂ average: 20 mL greater decline in children with AHR (all significant)	Short-term NO and NO₂ exposure caused significant acute FEV₁ declines. Children with airway hyperresponsiveness or persistent asthma experienced larger drops, though effects were also seen in children with normal airways.
Ouédraogo, 2021	Burkina Faso, Ouagadougou	Urban	Retrospective ecological	Children 0–15 yrs (*N* = 2102)	Jul 2019-Jun 2020	PM₂.₅ (above WHO guidelines) and PM₁₀ (within WHO guidelines)	Hospital visits for respiratory illness in children (outpatient vs. inpatient)	PM₂.₅ crude OR: 0.996 (95% CI 0.993–0.998, *p* = 0.003) — significant • PM₁₀ crude OR: 0.997 (95% CI 0.977–1.018, *p* = 0.802) — not significant	PM₂.₅ levels were frequently very high (up to 258.82 µg/m³). Increases in PM₂.₅ were linked to a slight shift toward outpatient rather than inpatient visits for respiratory disease. PM₁₀ showed no significant association. Weather factors confounded the relationship, while sociodemographic and medical factors did not.
Tamire, 2019	Ethiopia, Addis Ababa, Butajira	Urban, rural	Cross-sectional	Non-smoking women responsible for cooking (*N* = 545)	Mar-Aug 2016	Household air pollution from solid fuels	Respiratory symptoms; reduced FEV₁	• Respiratory symptoms: ~2× higher odds for women cooking inside the living area • FEV₁: Significantly lower among solid-fuel users (*p* < 0.05)	HAP from solid fuel use was strongly associated with more respiratory symptoms and reduced lung function in non-smoking Ethiopian women. Those cooking inside the home had double the odds of symptoms, highlighting elevated chronic respiratory risk—especially in rural settings.
Were, 2020	Kenya, Athi River Township	Industrialized suburb	Cross-sectional	Schoolchildren 9–14 yrs (*N* = 578)	Jan 2018-Jul 2019	PM₁₀ and PM₂.₅	Respiratory symptoms; lung function deficits	Elevated PM₁₀ and PM₂.₅ were strongly associated with lung function deficits and respiratory illness (RR/OR, *p* < 0.05).	Industrial-area schools had indoor PM levels consistently above WHO limits, while control schools had much lower levels. Higher PM exposure was linked to more respiratory symptoms and reduced lung function, especially during the wet season, when indoor concentrations were highest (indoor–outdoor ratios up to 2.40 for PM₁₀).

### Exposure Levels and Comparison with WHO Air Quality Guidelines

Measured air pollution levels frequently exceeded WHO – recommended standards across study sites. Indoor PM_2.5_ concentrations in biomass fuel-using households ranged from 95.69 to 177 µg/m³, substantially exceeding the WHO 24-hour limit of 25 µg/m³. In Burkina Faso, ambient PM_2.5_ concentrations ranged from 0.41 to 258.82 µg/m³, while PM_10_ levels (0.06–18.33 µg/m³) remained within WHO guidelines [10]. Kenyan schools near industrial areas exhibited indoor median PM_10_ levels of 60.8–269.1 µg/m³ during dry seasons and 52.8–232.3 µg/m³ during wet seasons, with PM_2.5_ values of 17.7–52.4 µg/m³ and 28.5–75.5 µg/m³, respectively [9]. Nearly all classrooms exceeded WHO recommended levels. In Nigeria’s Niger Delta, crude oil-impacted communities showed mean PM_2.5_, PM_10_, TVOCs, and HCHO concentrations consistently higher than less-impacted communities and frequently exceeding WHO and national standards [11]. Nitrogen oxide exposures in South African schools showed SO_2_ means of 8.7 ppb in southern (high-pollution) communities versus 1.9 ppb in northern communities, with PM_10_ maximum 24-hour concentrations approaching or exceeding 150 µg/m³ [12].

### Effects on Acute Respiratory Infections and Pneumonia

The association between indoor air pollution and ARI varied by pollutant type and study context. In Burkina Faso, biomass fuel use in indoor kitchens tripled the odds of ARI in children under five (OR = 3.79, *p* = 0.044) [10]. Living in houses with more than three bedrooms was protective (OR = 0.38, *p* = 0.013) likely due to the use of cleaner fuels for heating and cooking as well as better ventilation. In Nigeria, black carbon exposure was positively associated with pneumonia episodes (*p* = 0.026), with higher levels in cases (4,350 µg/m³) than controls (4,126 µg/m³) [13]. Interestingly, PM_2.5_ showed an inverse pattern, being higher in controls than cases at several time intervals (10, 15, and 20–24 h). The Malawian adult pneumonia study found no association between household air pollution exposure and pneumonia in either HIV-positive (adjusted Odds Ratio (aOR) = 1.00, 95% CI: 1.00-1.01, *p* = 0.141) or HIV-negative (aOR = 1.00, 95% CI: 0.99–1.01, *p* = 0.872) participants [14]. A potential limitation of the findings is that outdoor air pollution was not measured during the study. Given that participants were sampled from the same area, relatively similar or higher outdoor exposure level that rendered household air pollution exposure insignificant and potentially confounding observed results. However, chronic respiratory disease was strongly associated with pneumonia in both groups (HIV+: aOR = 28.07, 95% CI: 9.29–84.83; HIV-: aOR = 104.27, 95% CI: 12.86–852.35) (Table 3).

**Table 3:** Findings from the three studies that would have been included in the meta-analysis if there were more than 3 studies to include from the systematic review

Author	Location	Exposure–Outcome	Cases	Controls	Total	Unadjusted OR	Type of OR	95% CI	*P*-Value	Std. Mean Diff	SE	Subgroup Analyses
Jary et al., 2017	Malawi	Ambulatory PM exposure and pneumonia (overall)	145	253	398	2,08	Unadjusted	-	-	0,40	0,14	Overall
Jary et al., 2017	Malawi	Chronic respiratory disease and pneumonia (HIV-positive)	28	84	112	28,07	Adjusted	9,29–84,83	< 0,001	1,84	0,31	Stratified by HIV status
Jary et al., 2017	Malawi	Chronic respiratory disease and pneumonia (HIV-negative)	117	169	286	104,27	Adjusted	12,86–852,35	< 0,001	2,57	0,59	Stratified by HIV status
Hashazimari et al., 2022	Burundi	Landfill exposure and ARI (overall)	38,777	79,305	118,082	3,19	Unadjusted	-	-	0,64	0,00	Overall
Hashazimari et al., 2022	Burundi	Landfill exposure and ARI (exposed over 5)	9602	36,941	46,543	6,03	Unadjusted	-	-	0,99	0,01	Stratified by Age
Hashazimari et al., 2022	Burundi	Landfill exposure and ARI (exposed under 5)	29,175	42,094	71,269	1,71	Unadjusted	-	-	0,30	0,01	Stratified by Age
Mbeje et al., 2022	South Africa	Exposure-lung cancer Overall	75	159	234	-	-	-	-	-	-	Overall
Mbeje et al., 2022	South Africa	Tobacco smoking and lung cancer	49	57	106	2,86	Adjusted	1,21 − 6,77	0,017	0,58	0,24	Stratified by exposures
Mbeje et al., 2022	South Africa	Passive smoke exposure and lung cancer	41	47	88	3,28	Adjusted	1,48 − 7,30	0,004	0,66	0,22	Stratified by exposures
Mbeje et al., 2022	South Africa	History of lung disease and lung cancer	22	8	30	7,83	Unadjusted	3,29–18,7	< 0,001	1,14	0,24	Stratified by exposures
Mbeje et al., 2022	South Africa	Exposure to soot and lung cancer	7	2	9	8,08	Unadjusted	1,64–39,91	0,01	1,15	0,45	Stratified by exposures
Mbeje et al., 2022	South Africa	Exposure to iron and steel and lung cancer	8	2	10	9,37	Unadjusted	1,94–45,31	0,005	1,24	0,44	Stratified by exposures

### Effects on Lung Function

Studies measuring lung function demonstrated dose-response relationships between nitrogen oxides and spirometry parameters in South African schoolchildren. In South Africa, FEV_1_ declines ranged from 13 to 18 mL per IQR increase in NO_2_ and 14–23 mL per IQR increase in NO [12]. Effect modification by airway hyperresponsiveness was substantial: among 5-day average models, children with airway hyperresponsiveness experienced 20 mL and 30 mL greater drops in FEV_1_ per IQR for NO_2_ and NO, respectively, compared to those without hyperresponsiveness. Persistent asthmatics showed 40 mL reductions in FEV_1_ per IQR increase of NO_2_ and 70 mL reductions per IQR increase of NO. Notably, effects were observed even among children with normal airways. Ethiopian women cooking with solid fuels inside their living houses had nearly double the odds of developing respiratory symptoms compared to those using cleaner fuels (OR = 1.89, 95% CI: 1.11–3.24) [15]. FEV_1_ values were significantly lower among solid fuel users compared to cleaner energy users. The presence of permanent openings for ventilation was protective, though this effect was attenuated in multivariable analysis. Kenyan schoolchildren in industrialized areas exhibited high relative risks and odds ratios for respiratory diseases [9]. One school showed RR = 3.54 (95% CI: 1.62–7.71) and OR = 5.16 (95% CI: 2.05–14.83) for small airway obstruction during the dry season. Another school demonstrated consistent elevations in PM levels and associated lung function impairments.

### Hospital Visits and Healthcare Utilization

One study examined the relationship between particulate matter and paediatric hospital visits for respiratory diseases in Burkina Faso [10]. Rising PM_2.5_ concentrations were associated with slightly more outpatient than inpatient visits (OR = 0.996, 95% CI: 0.993–0.998, *p* = 0.003). However, PM_10_ increases were not significantly associated with hospital visits (OR = 0.997, 95% CI: 0.977–1.018, *p* = 0.802). Weather conditions, particularly relative humidity, confounded the PM_2.5_-hospital visit relationship. Females were more likely to be outpatients than inpatients (OR = 0.816, 95% CI: 0.678–0.982, *p* = 0.031). Underlying conditions including severe acute malnutrition, sickle cell disease, and heart disease favoured inpatient admissions over outpatient visits.

### Mortality Outcomes

The largest study examining mortality outcomes analysed 164,376 children under five across 14 sub-Saharan African countries using Demographic and Health Survey data [16]. Household air pollution exposure from biomass fuel use was associated with increased under-five mortality risk (adjusted OR = 1.33, 95% CI: 1.03–1.71). Kitchen location modified this association substantially: children from households cooking inside the home had higher mortality risk compared to those cooking in separate buildings (OR = 0.85, 95% CI: 0.73–0.98) or outside (OR = 0.75, 95% CI: 0.64–0.87). Breastfeeding demonstrated a strong protective effect, with breastfed children showing markedly lower mortality risk (OR = 0.09, 95% CI: 0.05–0.18) compared to non-breastfed children. The association between HAP and mortality was stronger in non-breastfed children, suggesting breastfeeding status as an important effect modifier. Female children had lower mortality risk than males, and higher maternal education and wealth index were associated with reduced mortality.

### Other Respiratory Outcomes

Two studies examined outcomes beyond acute infections, namely lung function, and mortality. A Nigerian study of crude oil-impacted communities found significantly higher prevalence of respiratory symptoms among children under five in oil-exposed areas compared to less-impacted communities [11]. Logistic regression revealed odds ratios ranging from 2.53 to 14.18 for various symptoms including cough (OR = 2.53, 95% CI: 1.55–4.13), difficulty breathing (OR = 6.32, 95% CI: 3.81–10.67), persistent nasal congestion (OR = 9.18, 95% CI: 5.41–15.54), and itchy nostrils (OR = 14.18, 95% CI: 8.13–24.73). An adult lung cancer case-control study in South Africa environmental tobacco smoke exposure (OR = 3.28, 95% CI: 1.48–7.30) as a significant risk factor.

### Birth Outcomes

One study examined associations between air pollution and low birthweight. An Ethiopian case-control study identified multiple risk factors for low birthweight, including environmental exposures [17]. Using firewood for cooking increased the odds of low birthweight (aOR = 2.7, 95% CI: 1.01–7.17), while kerosene use showed an even stronger association (aOR = 8.9, 95% CI: 2.54–31.11). Not having a separate kitchen room (aOR = 2.6, 95% CI: 1.36–4.85) was also associated with increased risk.

### Effect Modification and Vulnerable Populations

Several individual and contextual factors modified the associations between air pollution and respiratory outcomes. Mentz et al., 2019 showed airway hyperresponsiveness was a consistent effect modifier for lung function changes, with children having marked or probable airway hyperresponsiveness showing greater decrements compared to those without [12]. Asthma status similarly modified effects, with persistent asthmatics experiencing greater impacts than non-persistent asthmatics. This study in Durban showed greater impacts observed in southern (high level air pollution – industrialised area) versus northern communities (low-level air pollution, non-industrialised rural communities), potentially due to a lack of ground level air pollution monitoring. Age emerged as an important modifier. Children under five years were more affected by acute respiratory infections from waste dump exposure compared to older age groups. Malnutrition, measured by mid-upper arm circumference, increased susceptibility to pneumonia in Nigerian children [13]. Seasonal variations influenced both exposure levels and health effects. Pollutant concentrations were higher during rainy seasons in Nigeria, and associations between PM exposure and respiratory diseases were more prevalent during wet seasons in Kenya [9]. However, South African data collection phases showed higher pollutant levels in colder months. HIV status was a critical effect modifier in the Malawian pneumonia study, with analyses stratified by HIV status showing different patterns of association [14]. Chronic respiratory disease strongly predicted pneumonia in both HIV positive (aOR = 1.00; 95% CI 1.00–1.01, *p* = 0.141) and HIV-negative (PM aOR = 1.00; 95% CI 0.99–1.01, *p* = 0.872) participants. Reduced BMI and malnutrition were identified as important effect modifiers, particularly among HIV-positive individuals. Socioeconomic factors modified exposure-outcome relationships in multiple ways. Poverty influenced treatment seeking behaviour and exposure patterns. Higher maternal education and wealth index were associated with lower mortality risk in the 14-country analysis [16]. Living in rural areas increased the odds of low birthweight (aOR = 2.1, 95% CI: 1.04–4.33). Behavioural and household characteristics modified risks substantially. Kitchen location modified the HAP-mortality association, with cooking inside the home conferring higher risk. Breastfeeding status modified the HAP-mortality relationship, with stronger associations observed in non-breastfed children. Ventilation quality, measured by presence of permanent openings in cooking areas, was protective against respiratory symptoms. Living in houses with more bedrooms reduced ARI risk, likely through better ventilation.

### Quality of Included Studies

Of the 13 studies, four were cross-sectional [9, 11, 15, 16], one was a panel [12] study and one was an ecological study using hospital admission records [10]. Therefore, these studies did not have cases and controls and did not meet the criteria pertaining to case-control factors. Of the 6 case-control studies, three only included children in their target population [13, 18, 19]. As explained below, we were attempting to do a meta-analysis and therefore we only included case-control studies, with exception to the nine studies mentioned here. All four of the included case-control studies fully met the quality criteria for case-control studies.

### Towards a Meta-analysis

We had originally planned to conduct a meta-analysis as part of this systematic review; however after assessing the evidence, only four articles [8, 14, 17, 20] presented case-control studies (technically Mbeje et al. was an epidemiological study) that provided the necessary data. Given that three studies is insufficient for conducting a meta-analysis, as well as the range in morbidity outcomes reported in these studies, we did not pursue the meta-analysis. We present these findings here to support future meta-analyses of air pollution-related morbidity outcomes in Africa.

## Discussion

This review examines the impact of air pollutant exposures on respiratory health, low birth weight and mortality across Africa, with a focus on local environmental factors. We found 14 studies across 10 Sub-Saharan African countries. The main findings indicate diverse study designs, populations, and exposure assessment methods, encompassing ambient air pollutants such as PM₂.₅, PM₁₀, NO₂, NO, SO₂, household air pollution from biomass fuel use and even airborne microbial exposures. Temperature was measured in a few studies, ranging from 17 to 33 °C, though it was rarely an exposure of interest in air quality studies. Overall, these studies highlight the significant burden of both ambient and household air pollution on respiratory outcomes in vulnerable populations, particularly children under five and women exposed to indoor cooking emissions.

A substantial body of evidence highlights the harmful effects of PM on acute respiratory health. Elevated ambient concentrations of PM₂.₅ and PM₁₀ near crude oil spill sites increased the odds of respiratory symptoms in children under five by up to 14-fold [11]. Indoor exposure to BC, a toxic component of PM from incomplete combustion, was significantly associated with pneumonia episodes [13]. A meta-analysis of six studies across four sub-Saharan African countries (Ethiopia, Zimbabwe, Malawi, Ghana) involving 16,761 mother-child pairs found that indoor air pollution from biomass fuel use was associated with low birthweight (pooled OR = 1.74, 95% CI: 1.14–2.66). This represented a 74% increased risk of low birthweight for mothers living in households using biomass fuel compared to those without such exposure. Heterogeneity among studies was substantial (I²=81.8%, *p* < 0.000). Even in schools, PM levels frequently exceeded WHO guidelines, linking exposure to higher rates of respiratory disease and measurable lung function deficits among pupils [9]. These findings emphasize that both ambient and indoor PM sources are major drivers of acute respiratory morbidity.

The association between PM₂.₅ and mortality is strongly supported by an analysis showing that long-term exposure to PM₂.₅ significantly predicts all-cause, cardiovascular, and respiratory mortality, with effect estimates aligning with global meta-analyses [21]. Studies also highlight the interplay between air pollution and infectious diseases, demonstrating that higher ambient pollution is linked to increased tuberculosis incidence and other respiratory diseases, suggesting that pollution may impair host immune defences [22, 23]. Additionally, reproductive health impacts of household air pollution extend beyond low birth weight, encompassing outcomes such as stillbirths and preterm delivery, confirming HAP as a major environmental risk factor for maternal and neonatal health [24, 25].

HAP from biomass fuel use was linked to severe and chronic health outcomes, affecting neonatal survival and adult respiratory function. Pooled data across 14 sub-Saharan African countries showed that HAP exposure increased under-five mortality risk (aOR 1.33), with lower risk when cooking occurred outside the main living area [16]. Women exposed to HAP while cooking had twice the odds of respiratory symptoms and significantly reduced lung function (FEV₁) compared to cleaner fuel users [15].

Respiratory outcomes were also influenced by specific gaseous pollutants and environmental conditions. Short-term spikes in NO and nitrogen dioxide NO₂ caused immediate FEV₁ declines, particularly in children with airway hyperresponsiveness [12]. Proximity to unprotected landfills tripled the risk of ARI in exposed communities [8]. Protective factors, such as improved household ventilation, reduced ARI risk from indoor cooking [19]. These findings underscore the importance of multi-faceted mitigation strategies addressing chemical, microbial [7], and housing-related risk factors.

These findings underscore the urgent need for targeted policy and useful interventions to reduce both ambient and household air pollution exposure. Evidence supports housing-based strategies, such as moving cooking to separate buildings or outdoors, which significantly lower mortality and morbidity risks [16, 19]. Varying effects of pollutants like PM₂.₅ driving outpatient visits [10] and NO/NO₂ causing acute lung function declines [12] highlight the importance of monitoring a broad spectrum of pollutants, particularly in industrial and high-traffic urban areas. Although the current literature provides a strong foundation, heterogeneity in study design and reporting (with only three studies suitable for meta-analysis) underscores the need for standardized, longitudinal research in sub-Saharan Africa, with an emphasis on case control studies. Consistent methodologies will enable pooled analyses, refine regional air quality guidelines, and support more effective allocation of public health resources.

### Limitations

The review process entailed searches in two databases and could have included others such as Web of Science. However, we supplemented our search using Elicit since this was a rapid systematic review. We may have missed some articles that would have been included had we searched other databases. Also, we set out to only include case-control studies; however, since there were only three studies we did include in our systematic review other types of epidemiological studies, i.e., panel study, cross-sectional and ecological (hospital records) to provide a slightly wider view of the available literature.

## Conclusion

Collectively, this evidence demonstrates that ambient and household air pollution, combined with local environmental and socio-behavioural factors, significantly contribute to respiratory morbidity, impaired lung function, adverse birth outcomes, and under-five mortality in sub-Saharan Africa. Multi-faceted interventions, including cleaner fuel promotion, improved ventilation, and pollution control, are critical to reducing these burdens in vulnerable populations.

## Key References

Cohen AJ, Brauer M, Burnett R, Anderson HR, Frostad J, Estep K, et al. Estimates and 25-year trends of the global burden of disease attributable to ambient air pollution: an analysis of data from the Global Burden of Diseases Study 2015. The Lancet. 2017;389:1907–18. https://doi.org/10.1016/S0140-6736(17)30505-6

- ○ This manuscript emphasises the global burden of disease associated with exposure to air pollution.

Matthaios VN, Pope D, Koutrakis P, Olopade CO, North CM. The Struggle Against Air Pollution in African Megacities and the Hidden Problems for the Estimation of the Burden of Disease. Glob Chall. 2025;9:e00108. https://doi.org/10.1002/gch2.202500108

- ○ A synthesis of the challenges faced by Africa in terms of air pollution impacts on health.

## Supplementary Information

Below is the link to the electronic supplementary material.

Supplementary Material 1

Supplementary Material 2

## Data Availability

No datasets were generated or analysed during the current study.
