# Supplementary material for: Air Pollution and Heat Impacts on Respiratory Morbidity and Mortality Outcomes in Africa: A Systematic Review Towards a Meta-analysis
Source: Curr Environ Health Rep. 2026 Sep 3;13(1):39. doi: 10.1007/s40572-026-00561-7 (PMC13541811; doi:10.1007/s40572-026-00561-7)
Supplement: Supplementary file 1 [file 40572_2026_561_MOESM2_ESM.docx]

JBI Critical Appraisal Checklist for case control studies

Note: ✓ is Yes, x is no, n/a is not applicable; I is Include, E is Exclude.

|  | Article number | | | | | | | | | | | | | | |
| --- | --- | --- | --- | --- | --- | --- | --- | --- | --- | --- | --- | --- | --- | --- | --- |
|  | **1** | **2** | **3** | **4** | **5** | **6** | **7** | **8** | **9** | **10** | **11** | **12** | **13** | **14** |  |
| 1. Were the groups comparable other than the presence of disease in cases or the absence of disease in controls? | ✓ | n/a | ✓ | ✓ | n/a | ✓ | ✓ | n/a | ✓ | n/a | n/a | ✓ | n/a | ✓ |  |
| 2. Were cases and controls matched appropriately? | ✓ | n/a | ✓ | ✓ | n/a | ✓ | ✓ | n/a | ✓ | n/a | n/a | ✓ | n/a | ✓ |  |
| 3. Were the same criteria used for identification of cases and controls? | ✓ | n/a | ✓ | ✓ | n/a | ✓ | ✓ | n/a | ✓ | n/a | n/a | ✓ | n/a | ✓ |  |
| 4. Was exposure measured in a standard, valid and reliable way? | ✓ | ✓ | ✓ | ✓ | ✓ | ✓ | ✓ | ✓ | ✓ | X | ✓ | ✓ | ✓ | X |  |
| 5. Was exposure measured in the same way for cases and controls? | ✓ | n/a | ✓ | ✓ | n/a | ✓ | ✓ | n/a | ✓ | n/a | ✓ | ✓ | n/a | ✓ |  |
| 6. Were confounding factors identified? | ✓ | X | ✓ | ✓ | ✓ | X | ✓ | ✓ | ✓ | ✓ | ✓ | ✓ | ✓ | ✓ |  |
| 7. Were strategies to deal with confounding factors stated? | ✓ | X | ✓ | ✓ | ✓ | X | ✓ | ✓ | ✓ | ✓ | ✓ | ✓ | ✓ | ✓ |  |
| 8. Were outcomes assessed in a standard, valid and reliable way for cases and controls? | ✓ | n/a | ✓ | ✓ | n/a | ✓ | ✓ | n/a | ✓ | n/a | n/a | ✓ | n/a | ✓ |  |
| 9. Was the exposure period of interest long enough to be meaningful? | ✓ | ✓ | ✓ | ✓ | ✓ | ✓ | ✓ | ✓ | ✓ | ✓ | ✓ | ✓ | ✓ | ✓ |  |
| 10. Was appropriate statistical analysis used? | ✓ | ✓ | ✓ | ✓ | ✓ | ✓ | ✓ | ✓ | ✓ | ✓ | ✓ | ✓ | ✓ | ✓ |  |
| Overall appraisal | I | I | I | I | I | I | I | I | I | I | I | I | I | I |  |
